# Two new genera, *Hoffmannanthus* and *Jeffreycia*, mostly from East Africa (Erlangeinae, Vernonieae, Asteraceae)

**DOI:** 10.3897/phytokeys.39.7624

**Published:** 2014-07-18

**Authors:** Harold Robinson, Sterling C. Keeley, John J. Skvarla, Raymund Chan

**Affiliations:** 1 Department of Botany, MRC 166, National Museum of Natural History, Smithsonian Institution, P.O. Box 37012, Washington, DC., 20013-7012, USA; 2 Department of Botany, University of Hawaii, Manoa, 3190 Maile aWay, #101, Honolulu, Hawaii, 96822-2279, USA; 3 Department of Botany and Microbiology, and Oklahoma Biological Survey, University of Oklahoma, Norman, Oklahoma, 73018-6131, USA, deceased 2 March 2014

**Keywords:** Africa, Compositae, Erlangeinae, *Hoffmannanthus*, *Jeffreycia*, new genera, Vernonieae

## Abstract

Two genera of Vernonieae subtribe Erlangeinae with Type A pollen, 5-ribbed achenes, and blunt-tipped sweeping hairs on the styles are described as new, *Hoffmannanthus* with one species and with *Vernonia brachycalyx* O. Hoffm. as type, and *Jeffreycia* with five known species, with *Vernonia zanzibarensis* Less. as type. *Vernonia abbotiana* O. Hoffm. is neotypified and is an older name for *V. brachycalyx*.

## Introduction

The dismantling of the overly broad concept of *Vernonia* Schreb. in the Old World was begun by Robinson (1999a). In that study, the primary point was fully established, that there are no true members of the genus *Vernonia* native to the Eastern Hemisphere. The genus *Vernonia* is almost entirely North American (Robinson 1999b). Still, acceptance of segregate genera would inevitably depend on establishment of a reasonably complete coverage of the tribe, defining properly phyletic segregates, and discovery of reasonable characteristics by which the segregates can be distinguished. The present effort concentrates on a related group that contains a number of mostly African, woody perennial species of the subtribe Erlangeinae having 5-angled achenes, blunt-tipped sweeping hairs on the styles, and tricolporate type A pollen (Keeley and Jones 1977, 1979), also known as sublophate pollen (Skvarla et al. 2005). Two elements of this group are here described as new genera, *Hoffmannanthus* and *Jeffreycia*.

## Methods

Specimens examined were from the U.S. National Herbarium in Washington, DC. Microscopic structures were examined mostly using material mounted in Hoyer’s Solution (Anderson 1954). Preparation of pollen for scanning electron microscopy (SEM) consisted of acetolysis (Erdtman 1960) followed by the osmium-thiocarbohydrazide repeat procedure (Chissoe et al. 1995) and pulse sputter coating with a gold/palladium (60/40) target (Chissoe and Skvarla 1996). Examination was with a JEOL 880 (University of Oklahoma) SEM equipped with lanthanum hexaboride (LaB6) electron sources.

## Results and discussion

The genera *Hoffmannanthus* and *Jeffreycia*, described here as new, are evidently closely related, but of these only *Hoffmannanthus* has had its DNA sequenced (Keeley et al. 2007). The available DNA sequence results place *Vernonia brachycalyx* O. Hoffm., the type of *Hoffmannanthus*, in a subclade within the subtribe Erlangeinae (Keeley and Robinson (2009). According to the DNA sequence, that part of the subtribe contains *Vernoniastrum* H. Rob. (Robinson 1999a), and somewhat more distantly, *Orbivestus* H. Rob. (Robinson 1999a, 2009). Of these, *Vernoniastrum* differs by a more herbaceous habit, pointed sweeping hairs on the style branches, idioblasts of the achenes in transverse bands, and lophate, triporate pollen. *Orbivestus* has the same type of pollen as *Hoffmannanthus*, but the plant is more herbaceous, has heads in seriate or subscorpioid cymes, has nearly sessile T-shaped hairs on the stems, has strictly subimbricate and otherwise undifferentiated bracts in its involucres, has narrowly rhomboid raphids in the walls of the achenes, and has pointed tips on the sweeping hairs of the style branches. The sweeping hairs occur along the entire outer surface of the style branches. Sequence data is lacking for *Jeffreycia*, but on the basis of structural evidence, *Jeffreycia* is considered closer to *Hoffmannanthus* than *Vernoniastrum* or *Orbivestus*. As shown in the review by Herz (1996), *Jeffreycia* (as *Vernonia zanzibarensis*) and *Hoffmannanthus* (as *Vernonia brachycalyx*) also share an unusual type of glaucolide derivative that has otherwise been reported only from *Bothriocline* (as *Bothriocline amplifolia*), all three genera evidently members of the Erlangeinae in the strict sense.

The typical element of the subtribe Erlangeinae with the genera *Erlangea* Sch. Bip., *Bothriocline* Oliv. ex Benth., and *Cyanthillium* Blume consists of herbaceous plants with mostly lophate, triporate pollen, symmetrically T-shaped hairs, and sharply pointed sweeping hairs on the styles. In contrast, the two genera described herein are shrubbier or weakly arborescent with sublophate, tricolporate pollen having a continuous perforated tectum between the colpi, simple or asymmetrical non-T-shaped hairs, and blunt tips on the sweeping hairs.

**Figure 1.**
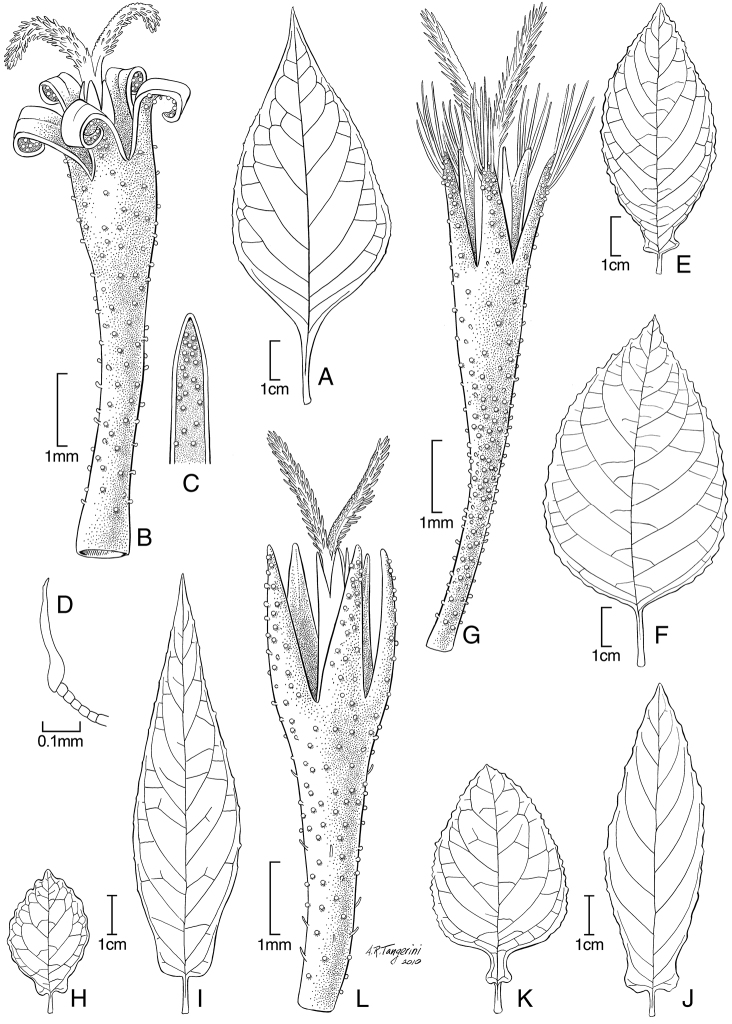
**A–D** *Hoffmannanthus abottianus* O. Hoffm. **E–G** *Jeffreycia zanzibarensis* (**E** form with panduriform leaves **F, G** typical form) **H** *Jeffreycia hildebrandtii* **I** *Jeffreycia amaniensis* **J** *Jeffreycia usambarensis* **K, L** *Jeffreycia zeylanica*. **A, F, H–K** leaf **B, G, L** corolla **C** lobe of corolla **D** stem hair (**A, D** from Kenya, *Gichuon 10*, US **B, C** from Ethiopia, *Burger 1816*, US **E** from *Rulangaranga et al. 83*, US **F, G** from Tanzania, *Faulkner 3866*, US **H** from Tanzania, *Stuhmann 7537*, US **I** from Tanzania, *Peter O III 7*, US **J** from Tanzania, *Peter O IV 15*, US **K, L** from Sri Lanka, *Silva 4*, US).

The two new genera, *Hoffmannanthus* and *Jeffreycia* (Fig. 1), share one feature found in many Old World Vernonieae, namely the sweeping hairs which are restricted to the branches of the style and do not extend onto the upper shaft, a feature otherwise a defining characteristic of the tribe Vernonieae and the subfamily Cichorioideae. *Jeffreycia* may be the most extreme in this character, with the sweeping hairs usually failing to even reach the bases of the style branches.

These genera are treated here together to allow a more effective direct comparison. The most obvious differences between *Hoffmannanthus* and *Jeffreycia* are found in the hairs of the stems, the shape of the leaves, and in details of the corolla lobes. The hairs on the stems of *Hoffmannanthus* have a rather long, uniseriate multicellular stalk with an elongate, asymmetrically mounted horizontal cap cell at the tip, these hairs being what could be called L-shaped (Fig. 1D). In contrast, the hairs on the stems of *Jeffreycia* species are simple and unbranched. The leaves of *Hoffmannanthus* have long petioles below the distinct basal acumination of the blade, and have no auricles on the blades (Fig. 1A). The leaves of all *Jeffreycia* except the typical variant of the type species, *Jeffreycia zanzibarensis* (Less.) H. Rob., S. Keeley & Skvarla have short petioles and blades with auricles projecting laterally at the base (Fig. 1E, F, I–K). The corolla lobes in *Hoffmannanthus* are oblong-triangular, and usually recurved at maturity (Fig. 1B, C). *Jeffreycia* has corolla lobes that are strictly lanceolate, the sides not parallel in any part, but evenly convergent from the base to the tip (Fig. 1G, L). The lobes are erect though sometimes withered when dry, but never recurved. A less obvious difference is the tendency for the pappus bristles in *Hoffmannanthus* to be sordid or even rufous and broader in the distal half, while those of *Jeffreycia* tend to be white and narrowed above.

Jones (1981) placed the type species of *Hoffmannanthus*, *Vernonia brachycalyx* O. Hoffm., in his *Vernonia* subsect. *Strobocalyx* S.B. Jones, among species now placed in the mostly African *Gymnanthemum* Cass. and in the mostly Asiatic and Malaysian genus *Strobocalyx* (Blume ex DC.) Spach. Jeffrey (1988) placed the species in his group 2 subgroup B, in an aggregate 3, distinguished by its persistent involucral bracts, 5-angled achenes, and ovate to cordate, non-panduriform leaves. *Vernonia brachycalyx* was not treated in the first effort to resolve palaeotropical Vernonieae by Robinson (1999a). Relationships of the species of *Hoffmannanthus* are considered to be particularly close to *Jeffreycia* which was placed by Jeffrey (1988) in his group 2, subsection B, aggregate 2. The present study shows that the two genera share Type A sublophate pollen, 5-angled achenes with short raphids, and blunt sweeping hairs on the style branches. Like many of the Old World Vernonieae, the sweeping hairs in *Hoffmannanthus* are lacking on the upper shaft of the style, but unlike most *Jeffreycia*, are not lacking on the bases of the style branches. Both genera are most common in east Africa. It is concluded that the two genera described here as new are closely related to each other but distinct.

The second genus treated here, *Jeffreycia*, includes three of the species mistakenly placed in *Gymnanthemum* in the subtribe Gymnantheminae by Robinson (1999a), a genus from which the present group is now seen to be subtribally distinct. Among the most obvious differences are the presence in *Gymnanthemum* of a broad abaxial shield in the involucral bracts and the tendency for the inner involucral bracts to be deciduous in *Gymnanthemum*, instead of persistent as in *Jeffreycia*.

A genus that is possibly closely related to *Jeffreycia* is the recently described *Uniyala* H. Rob. & Skvarla of India and Sri Lanka (Robinson and Skvarla 2009) with which *Jefferycia* scarcely overlaps geographically, only through its one species in Sri Lanka. While superficially similar, *Uniyala* has a shrubbier habit with closely spirally inserted leaves, non-panduriform bases of the blades, elongate raphids and thinner walled cells in the achene wall, and short tubes on the corollas and corolla lobes that are not strictly triangular.

**Figure 2.**
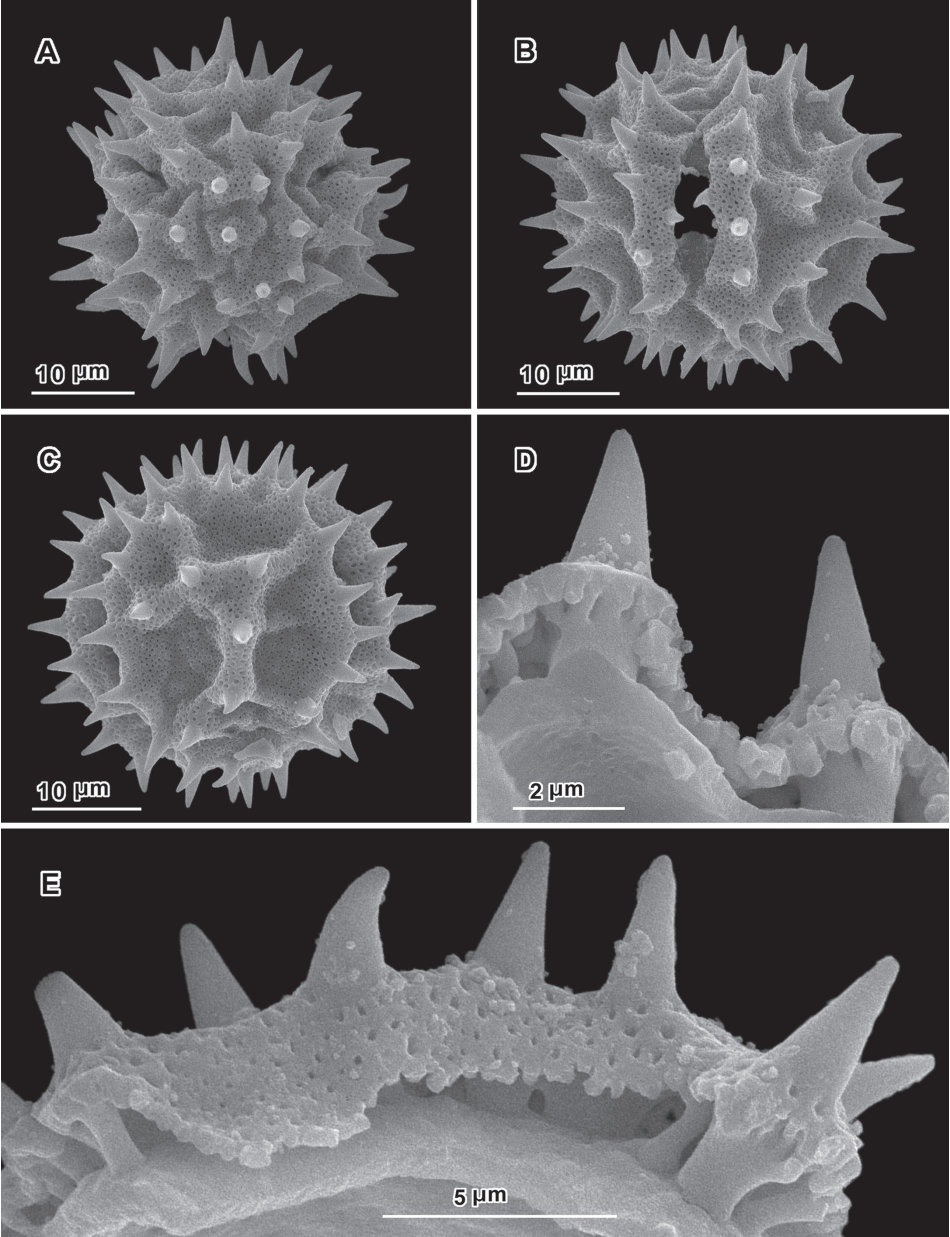
Scanning electron micrographs of *Hoffmannanthus abbotianus* pollen (Kenya, *Gichuon 10*, US). **A** polar view **B** equatorial view **C** lateral view **D, E** views of fractured grains.

**Figure 3.**
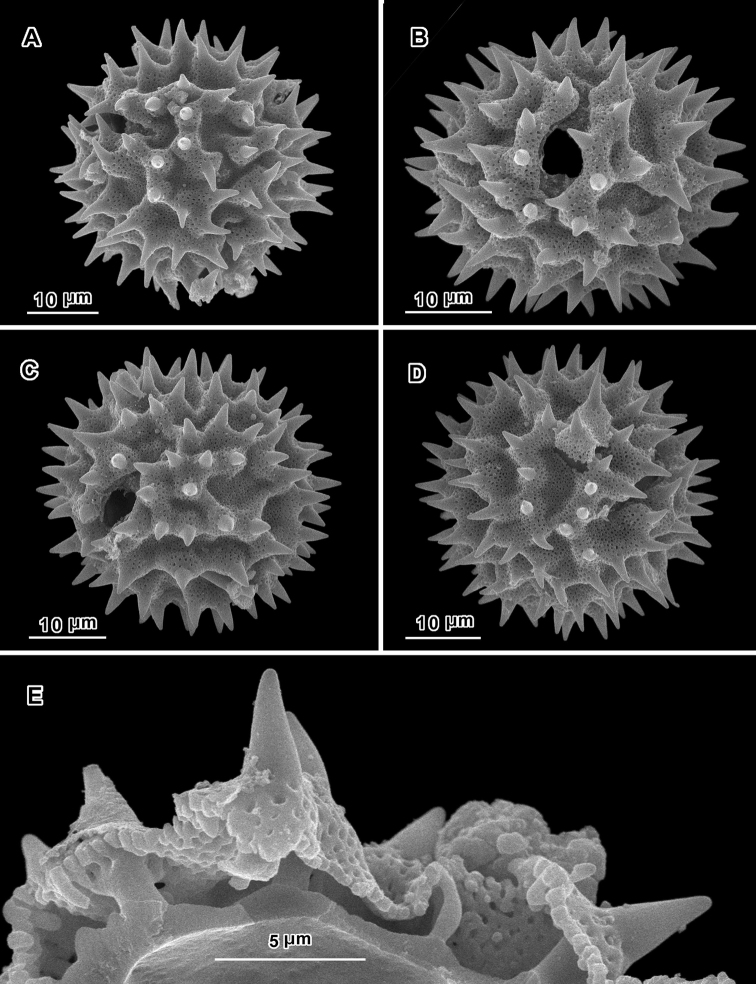
Scanning electron micrographs of *Jeffreycia zanzibarensis* pollen (Tanzania, *Faulkner 3866*, US). **A** polar view **B** equatorial view **C** oblique lateral view **D** lateral view **E** view of fractured grain.

**Figure 4.**
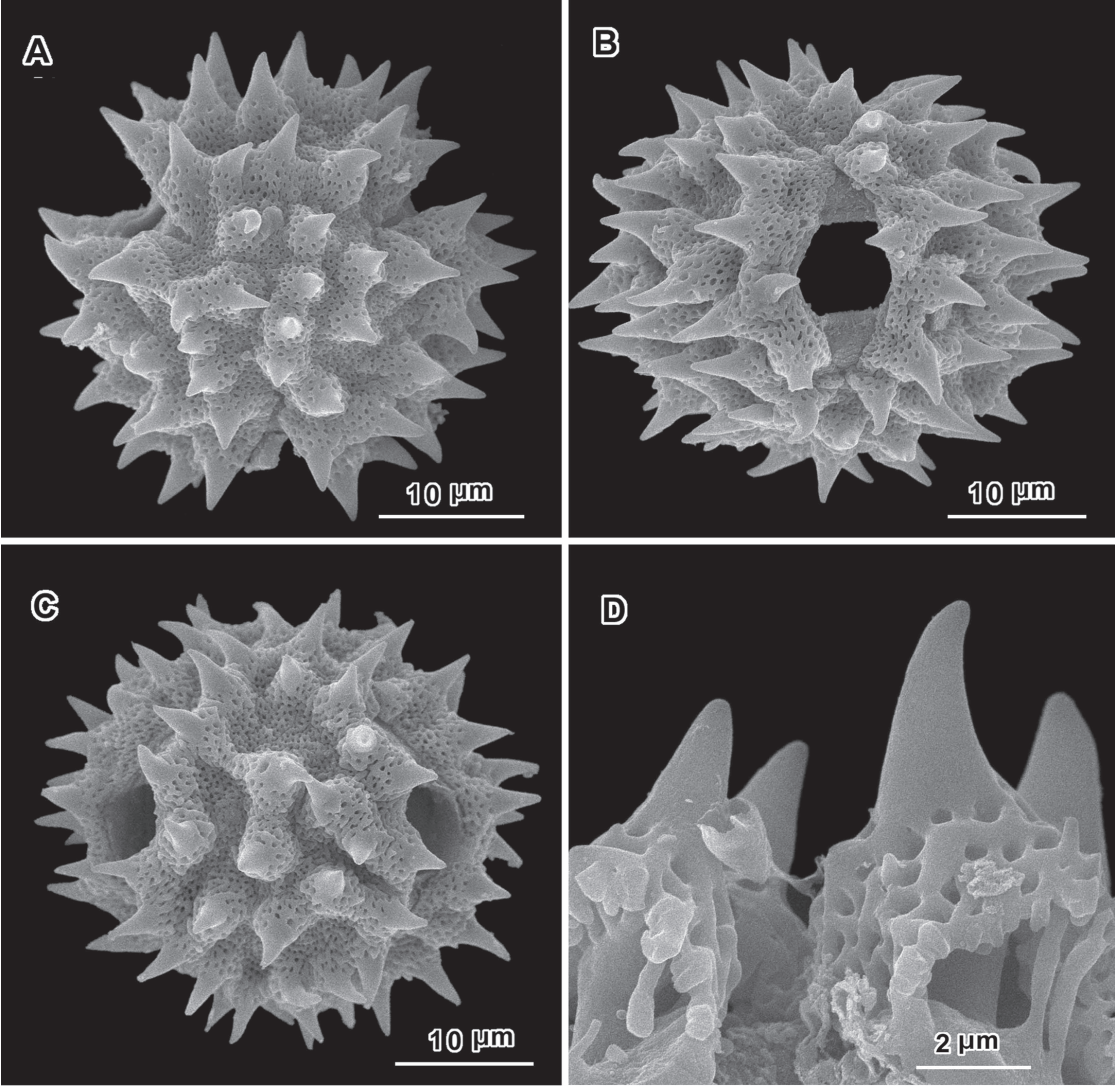
Scanning electron micrographs of *Jeffreycia zeylanica* pollen (Ceylon, *K. Wirawan 695*, US). **A** polar view **B** equatorial view **C** lateral view **D** fractured grain.

**Figure 5.**
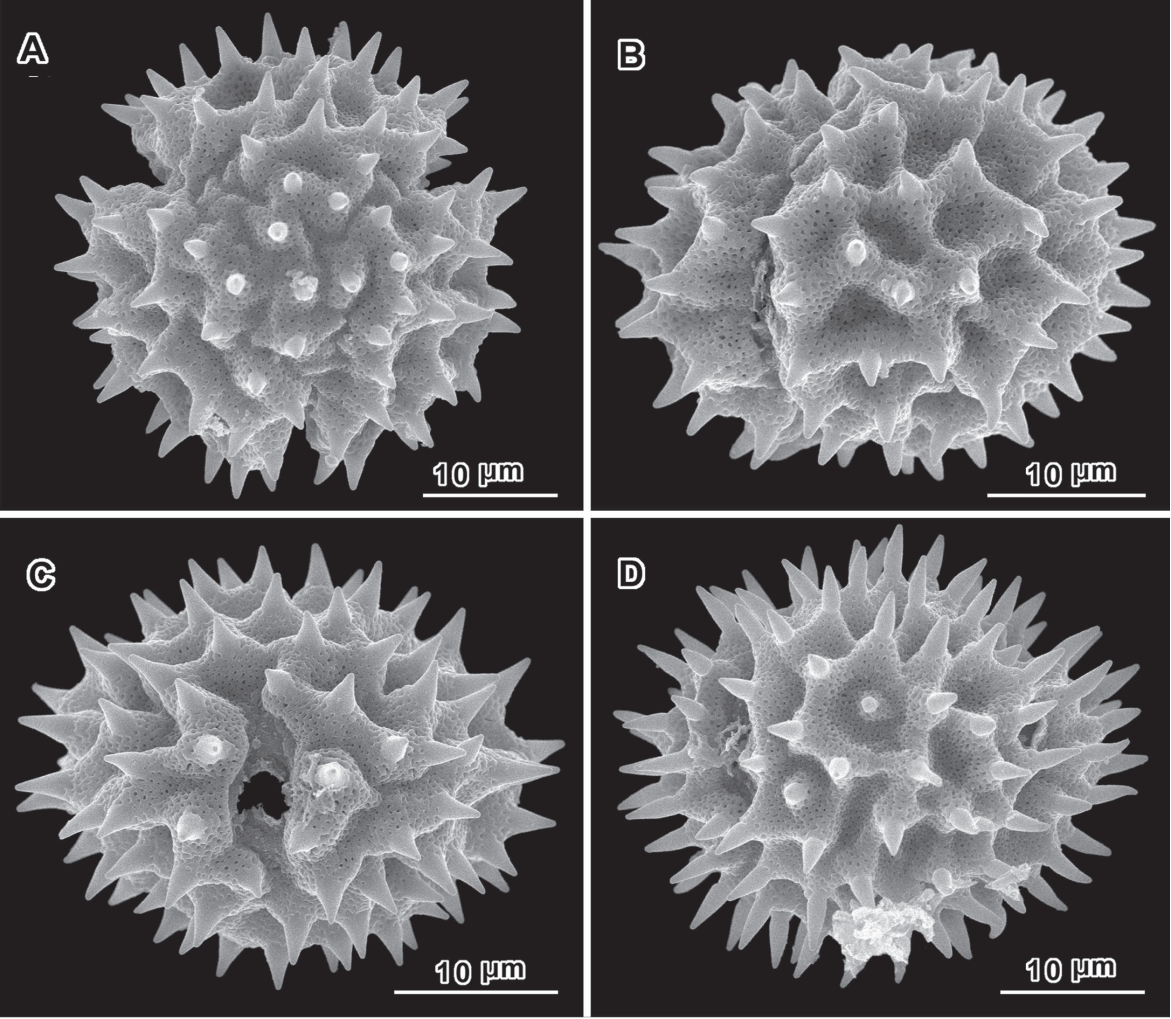
Scanning electron micrographs of *Jeffreycia* pollen: **A, B** J. usa*mbarensis* (Ost-Africa, *A. Peter 0 IV 15*, US) **C, D** J. hildeb*randtii* (Kenya, *J. Lauranos 12468*, US). **A** polar view **B, D** lateral view **C** equatorial view.

An apparent additional distinction between *Uniyala* and both *Jeffreycia* and *Hoffmannanthus* is the pollen. In all three genera, the pollen is approximately the same size, ca. 30 µm in diam. when dry, up to 50 µm in diam. in fluid, type A tricolporate or sublophate with a continuous perforated tectum between the colpi. However, in *Uniyala* the pollen has incipient muri more defined with fewer, larger incipient lacunae (Robinson and Skvarla 2009) than in the present group, where muri are obscure and the incipient lacunae are small and more numerous (Figs 2–5).

The generic segregates of *Vernonia* in tropical Africa are only partially resolved. A treatment of all but two species in Southern Africa is nearly complete, but it does not include any close relatives of the two genera described here. Nevertheless, the presently recognized tropical African genera that have previously been placed in *Vernonia* can be partially distingushed by the following key. Its utility is limited by the number of segregates of tropical African Vernonieae that remain untreated.

### Identification key to the segregate genera of Vernonieae of East Africa

**Table:** 

1	Leaves triplinervate; corollas sometimes yellow	*Distephanus* Cass.
–	Leaves with pinnate venation; corollas only purplish, bluish or white	2
2	Strictly herbaceous	3
–	Weakly to strongly woody, prostrate shrubs to small trees	7
3	Perennial herbs with root crown with densely pilose apex; often flowering before leaves appear	*Vernonella* Sond.
–	Annual or perennial herbs with rootstock not densely pilose at apex; flowers usually appearing after leaves	4
4	Mostly small herbs with pollen either sublophate, triporate or pantoporate	(most Erlangeinae and Centrapalinae) *Cabobanthus* H. Rob., *Centrapalus* Cass., *Cyanthillium* Blume, *Lettowia* H. Rob. & Skvarla, *Oocephala* (S.B. Jones) H. Rob., *Orbivestus* H. Rob., *Parapolydora* H. Rob., *Polydora* Fenzl, *Vernoniastrum* H. Rob.
–	Pollen lophate with three fully developed colpi	5
5	Weak herbs with involucral bracts bearing smooth broad shields abaxially, not marginally toothed or apically appendaged	*Anathura* H. Rob. & Skvarla
–	Coarse herbs to subshrubs; involucral bracts marginally toothed or apically appendaged	(Linziinae) 6
6	Corollas with lobes not longer than the throat; pappus segments flattened; muri of pollen echinate	*Baccharoides* Moench
–	Corollas with lobes longer than throat; pappus of capillary segments; muri of pollen psilate	*Linzia* Sch.Bip. ex Walp.
7	Involucral bracts broad, smooth abaxially with broad median shield; inner bracts often deciduous	*Gymnanthemum*
–	Involucral bracts oblong-lanceolate to linear-lanceolate, without broad smooth shield abaxially; inner bracts persistent	8
8	Pollen lophate, with little or no perforated tectum	*Ambassa* Steetz
–	Pollen sublophate, with continuous perforated tectum in intercolpi	9
9	Leaf blades tapering into petiole at base; corolla lobes oblong-lanceolate, recurved; hairs of stems with asymmetric cap cells	*Hoffmannanthus*
–	Leaf blades usually with basal auricles; corollas with erect lanceolate lobes; hairs of stems simple	*Jeffreycia*

## Taxonomic treatment

### Hoffmannanthus

Taxon classificationPlantaeAsteralesAsteraceae

H. Rob., S.C. Keeley & Skvarla gen. nov.

urn:lsid:ipni.org:names:77140770-1

#### Type.

*Vernonia brachycalyx* O. Hoffm.

Scrambling shrubs; stems slender with solid pith, somewhat deflected at nodes in upper part of vegetative plant and in inflorescence; hairs of stems L-shaped, with long, multicellular, uniseriate stalk and elongate, horizontal cap cell mounted near one end. Leaves alternate, petioles slender and 7–15 mm long below basal acumination of blade; blades ovate, 6–7 times longer than petiole, 5–10 cm long, 1.5–5.0 cm wide, base broadly obtuse to short-acute, narrowly acuminate at petiole, margins remotely denticulate to nearly entire, apex scarcely to gradually acuminate, surfaces pilosulous and with glandular dots, hairs sparser above, dense on larger veins; secondary veins pinnate, with ca. 6 weak secondary veins on each side of midrib, spreading at ca. 40-45° angles. Inflorescences broadly corymbiform, with branches elongate, mostly with small or insignificant bracteoles at bases; peduncles 2–30 mm long. Heads campanulate; involucre much shorter than florets at maturity; involucral bracts in 2–3 series, persistent, oblong-lanceolate, with acute to short-acuminate tips, puberulous outside, pale at base, midvein broadly greenish, percurrent at tip, lateral margins thinly membranous; receptacle scarcely convex, epaleate, epilose. Florets ca. 15 in a head, homogamous, bisexual; corollas violet to purple, narrowly funnelform, with long basal tube, throat short, lobes narrowly oblong-lanceolate, with glandular dots outside; anthers with triangular apical appendages; base of style slightly enlarged, style shaft glabrous, sweeping hairs on style branches elongate with rounded or blunt tips. Achenes 5-angled, with some glandular dots and short setulae, surface with sparse idioblasts and inner layer with small subquadrate or rounded raphids; pappus pale to sordid or rufous, 2 series, inner pappus of many capillary bristles that are slightly broader in distal half, outer pappus of short narrow scales. Pollen grains 40 µm in diam., Type A, sublophate. 2n = 20 (Jones 1982, as *Vernonia brachycalyx*).

#### Etymology.

The name *Hoffmannanthus* is considered appropriate, since both of the older species names featured here were published by Hoffmann (1894, 1895).

#### Number of species.

The genus contains the single species.

### Hoffmannanthus abbotianus

Taxon classificationPlantaeAsteralesAsteraceae

(O. Hoffm.) H. Rob., S.C. Keeley & Skvarla comb. nov.

urn:lsid:ipni.org:names:77140772-1

Vernonia abbotiana O. Hoffm., Bot. Jahrb. Syst. 20: 221. 1894. Type: Tanzania, Kilimandjaro, *Abbot 1890* (holotype B destroyed). Neotype (selected here): Tanzania, Kwa Mshusa, May 1893, *Holst 9096* (US, lectotype of *Vernonia brachycalyx* O. Hoffm.).
Vernonia brachycalyx O. Hoffm. in Engler, Pflanzenw. Ost-Afr. C: 405. 1895. Type: Tanzania Kwa Mshusa, *Holst 9096* (syntype B destroyed; lectotype US, selected here, isolectotypes BM, K).
Vernonia meiocalyx S. Moore, J. Bot. 38: 155. 1900. Type: Kenya, *Delamere s.n.* (syntypes BM).
Vernonia hoffmanniana S. Moore, J. Bot. 38: 156. 1900, nom. nud.
Vernonia jodopappa Chiov., Racc. Bot. Miss. Concol.: 60. 1935, nom. illeg., non Sch. Bip. 1845. *Vernonia jodopapposa* Lanza, Miss. Biol. Borana, Racc. Bot. Angiosp.-Gymnosp.: 244. 1939. Type: Kenya, Nyeri, *Balbo 428* (holotype TOM, isotype FI).

#### Distribution.

The species occurs from Ethiopia, Congo and Uganda in the north to Angola, Malawi and Zambia to the south.

#### Notes.

**Figure 6.**
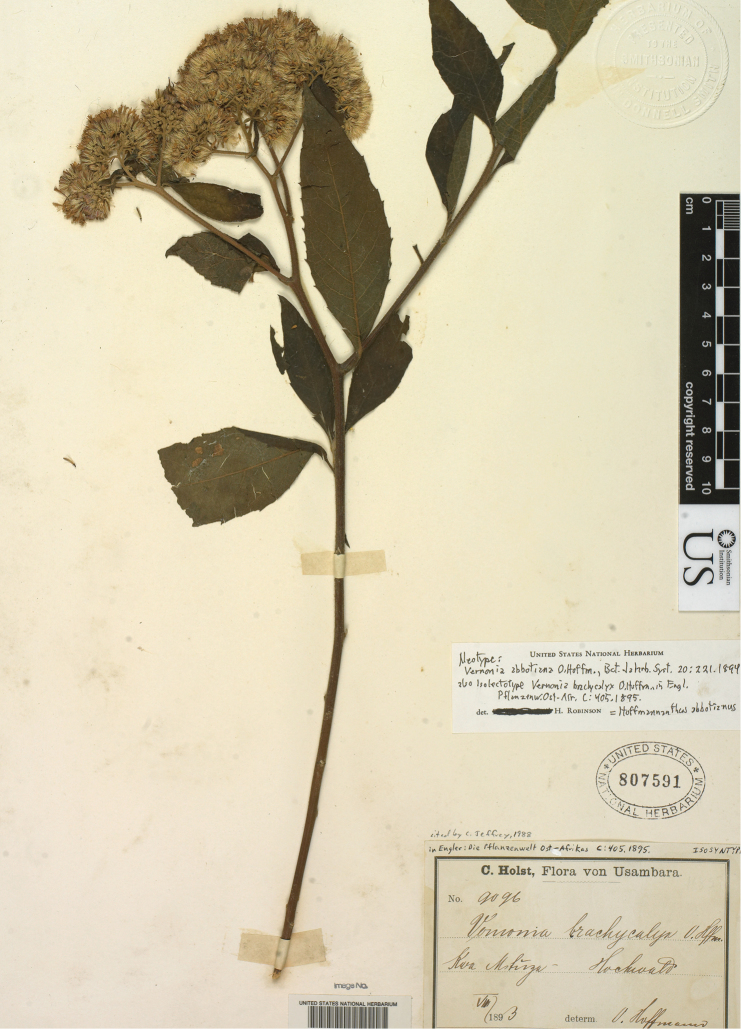
Neotype of *Vernonia abbotiana* O. Hoffm. and lectotype of *Vernonia brachycalyx* O. Hoffm.

The type specimen of *Vernonia abbotiana* O. Hoffm. was destroyed in Berlin during the Second World War, and the species was treated by Jeffrey (1988) as one of group titled, “taxa insufficiently known”. No duplicates of the type are known. Nevertheless, a specimen, *A. Peter OI 119*, from Tanzania, Usambara, collected May 25, 1914, identified as *Vernonia abbotiana*, was deposited in the US. National Herbarium. Although not a type, the specimen led to a careful comparison with the original description of the species (Hoffmann 1894). The specimen was finally recognized as a *Vernonia brachycalyx* with unusually long peduncles, but almost certainly fitting the description of *Vernonia abbotiana* in all details except the peduncles and the supposedly deciduous inner involucral bracts. The inner involucral bracts of *Vernonia brachycalyx* are not deciduous, but the involucre is short, giving the appearance of a missing inner series. As for the density of the inflorescence in *Vernonia abbottiana*, the original author, Hoffmann (1894) compared his species with *Vernonia livingstoniana* Oliv. & Hiern, which is a synonym of *Gymnanthemum thomsonianum* (Oliv. & Hiern) H. Rob. The latter species is not a close relative, but the reference to it in the original description indicates the kind of dense inflorescence. Such a dense inflorescence is unlike that in the Peter specimen, but it is very like typical material of *Vernonia brachycalyx* with which the Peter specimen is now identified. The identification might never have been made without the advent of the Peter specimen, but names such as *Vernonia abbotiana*, dating from comparatively early in the study of tropical Africa, do need to have their identity resolved by some means, in this case by neotypification. Personally, there is no doubt of the identification provided here, and a neotype, that is an isolectotype of *Vernonia brachycalyx* at the US National Herbarium is selected (Fig. 6), a specimen that matches the denser form of the inflorescence that is indicated by Hoffmann (1894).

### Jeffreycia

Taxon classificationPlantaeAsteralesAsteraceae

H. Rob., S.C. Keeley & Skvarla gen. nov.

urn:lsid:ipni.org:names:77140771-1

#### Type.

*Vernonia zanzibarensis* Less.

Small to moderate-sized; branching, often scrambling shrubs; stems woody, with narrow solid pith; hairs simple, without cap-cells, sometimes forming loose tomentum. Leaves alternate; petioles distinct, short to elongate; blades ovate to elliptic or panduriform usually with basal auricles, abruptly delimited from petiole at the base, 2.5 to ca. 11 cm long, ca. 1.5–7.5 cm wide, margins crenate or serrate, apices acute to scarcely acuminate, rarely obtuse, upper surface sparsely pilosulous to hispidulous, lower surface sparsely pilosulous to tomentellous, with many glandular dots; secondary veins 4–6 on each side, with unusual somewhat meandering course, spreading at 45–60° angles. Inflorescences terminal, with branches alternate and usually ascending at 30° angles or less, usually with minute bracteoles, sometimes primary bracteoles larger and foliiform; heads crowded at ends of longer branches, with distinct short peduncles; involucral bracts persistent, subimbricate in ca. 4–5 series or with differentiated long, linear-lanceolate basal bracts, bracts, except at base, smooth outside, without median keel; receptacle scarcely convex, epaleate, epilose, with proturberant scars; florets 5–40 in a head; corollas purplish, 5–11 mm long, with some glandular dots outside, few or no hairs below tips, basal tube slender, half as long as the corolla, throat half as long as the limb, ca. as long as the lobes, lobes strictly narrowly lanceolate, with sides straight from base to apex, erect, not recurving, sometimes with stiff hairs at tip; anther thecae without glands, calcarate at base, with narrow tails; endothecial cells without obvious nodes; apical appendages narrowly lanceolate; style with basal node; sweeping hairs with blunt tips, restricted to branches, often lacking for some distance above bases of branches. Achenes 2–4 mm long, with 4 or 5 poorly differentiated angles, with or without glands or setulae, with scattered idioblasts on surface sometimes in vertical series, inner cells of achene wall with distinct firm cell walls, containing small subquadrate raphids; carpopodium stopper-shaped or somewhat turbinate and asymmetrical, with many series of subquadrate, thick-walled cells; pappus white, with inner series capillary, often deciduous, 4.5–7.0 mm long, gradually narrowed to tips, somewhat flattened on outer surface; outer series of short persistent scales, minute to 0.5 mm long. Pollen ca. 40 µm in diam. in fluid, sublophate, tricolporate, with perforated tectum continuous between colpi.

#### Etymology.

The new genus, *Jeffreycia*, honors the author of the study of the Vernonieae of East Tropical Africa (Jeffrey 1988) whose work has been one of the most helpful in resolving the tribe in Africa.

#### Number of species.

Five species are currently placed in the genus.

In addition to the species listed below, Jeffrey (1988) included another three species in his aggregate, *Vernonia bruceae* C. Jeffrey, *Vernonia stuhlmanii* O. Hoffm., and *Vernonia fischeri* O. Hoffm., but these have not been seen in this study and therefore are not included in the new genus. Of these, *Vernonia fischeri* O. Hoffm. (1895) and *Vernonia stuhlmannii* O. Hoffm. (1898) are described with leaf bases truncate to subcordate, and both species are probably members of *Jeffreycia*, distinguished from the others by the appendages on the tips of their involucral bracts. However, *Vernonia brucaea* is described with “foliis ellipticis vel lanceolatis basi late cuneatis vel rotundatis“. Not stated is whether that leaf base is as abrupt at the insertion on the petiole as in all the species of *Jeffreycia* recognized here, and any close relationship to *Jeffreycia* is doubtful.

#### Notes on morphology.

Regarding the shape of the leaf base, while it is similar to cordate, Jeffrey (1988) refers to it as panduriform. The auricles result mostly from a constriction above the base of the leaf blade. This character is lacking only in those specimens of *Vernonia zanzibarensis* Less. that have longer petioles. Some specimens combine long hairs at the apices of the corolla lobes as in *Vernonia zanzibarensis* with panduriform bases on short-petiolate leaves, and it is apparently plants like these that have been interpreted by Jeffrey (1988) as hybrids between that species and *Vernonia hildebrandtii* Vatke. However, it is possible that such leaf blades are just a variant of *Vernonia zanzibarensis* that has reverted to or retained the leaf form that is characteristic of all the other members of the genus.

### Jeffreycia amaniensis

Taxon classificationPlantaeAsteralesAsteraceae

(Muschl.) H. Rob., S.C. Keeley & Skvarla comb. nov.

urn:lsid:ipni.org:names:77140773-1

Vernonia amaniensis Muschl., Bot. Jahrb. Syst. 46: 78. 1911. Type: Tanzania, Amani, *Zimmerman & Warnecke 90* (B destroyed, isotypes BM, K).

#### Distribution.

Tanzania.

### Jeffreycia hildebrandtii

Taxon classificationPlantaeAsteralesAsteraceae

(Vatke) H. Rob., S.C. Keeley & Skvarla comb. nov.

urn:lsid:ipni.org:names:77140774-1

Vernonia hildebrandtii Vatke, Oesterr. Bot. Z. 25: 323. 1875. *Gymnanthemum hildebrandtii* (Vatke) H. Rob., Proc. Biol. Soc. Washington 112(1): 241. 1999. Type: Tanzania, Zanzibar, *Hildebrandt 1020* (B destroyed, isotype K).
Vernonia taylorii S. Moore, J. Bot. 38: 154. 1900. Type: Kenya, Rabai Hill, *Taylor s.n.* (holotype BM).

#### Distribution.

Kenya, Somalia, Tanzania.

### Jeffreycia usambarensis

Taxon classificationPlantaeAsteralesAsteraceae

(O. Hoffm.) H. Rob., S.C. Keeley & Skvarla comb. nov.

urn:lsid:ipni.org:names:77140775-1

Vernonia usambarensis O. Hoffm., Bot. Jahrb. Syst. 20: 220. 1894. Type: Tanzania, Kwa Mshusa, *Holst 9146* (syntype B destroyed, isosyntype K) & Tanzania, Mlalo, *Holst 129, 203* (syntypes B destroyed).

#### Distribution.

Tanzania.

### Jeffreycia zanzibarensis

Taxon classificationPlantaeAsteralesAsteraceae

(Less.) H. Rob., S.C. Keeley & Skvarla comb. nov.

urn:lsid:ipni.org:names:77140776-1

Vernonia zanzibarensis Less., Linnaea 6: 637. 1831. *Gymnanthemum zanzibarensis* (Less.) H. Rob., Proc. Biol. Soc. Washington 112(1): 243. 1999. Type: “Bojer in insula Zanzebar (v. sp. in hrb. Horn.)” (Lessing 1831).

#### Distribution.

Kenya, Tanzania.

### Jeffreycia zeylanica

Taxon classificationPlantaeAsteralesAsteraceae

(L.) H. Rob., S.C. Keeley & Skvarla comb. nov.

urn:lsid:ipni.org:names:77140777-1

Eupatorium zeylanicum L., Sp. Pl.: 837. 1753. *Vernonia zeylanica* (L.) Less., Linnaea 4: 344. 1829. *Gymnanthemum zeylanicum* (L.) H. Rob., Proc. Biol. Soc. Washington 112(1): 243. 1999. Type: Herb. Hermann 4: 22 (lectotype BM000628096, selected here). The previous lectotype designation (Grierson 1980: 131) refers to at least three (possibly five) specimens, and this choice is narrowed here.

#### Distribution.

Sri Lanka.

### Key to the five species presently placed in the genus *Jeffreycia*

**Table:** 

1	Heads with 5–10 florets; with only rather short involucral bracts at base; corollas 5–6 mm long; leaf blades with crenate margins	2
–	Heads with 20–40 florets; with elongate filiform bracts at base; corollas 7–11 mm long; leaf blades with serrate margins	3
2	Undersurfaces of leaves and branches of inflorescence with short hispidulous pubescence; heads with ca. 10 florets	*Jeffreycia hildebrandtii*
–	Undersurfaces of leaves and branches of inflorescence with long hairs forming tomentum; heads with ca. 5 florets	*Jeffreycia zeylanica*
3	Corollas with cluster of long stiff hairs at tips of lobes; leaf blades usually ovate with margins closely serrate; with small bracteoles in the inflorescences	*Jeffreycia zanzibarensis*
–	Corollas lacking cluster of long hairs at tips of lobes; leaf blades oblong or elliptical; with remotely serrate margins; inflorescences with large foliiform primary bracteoles	4
4	Peduncles with appressed stiff hairs; leaf blades shortly pubescent below; inner involucral bracts to ca. 8 mm long	*Jeffreycia amaniensis*
–	Peduncles with mostly spreading, crisped hairs; leaf blades crispate pubescent below, somewhat obscurely pubescent on lamina surface; inner involucral bracts ca. 6 mm long	*Jeffreycia usambarensis*

## Acknowledgements

We wish to thank Alice Tangerini, Staff illustrator of the Department of Botany, National Museum of Natural History for the line drawings of the leaves and corollas of *Hoffmannanthus* and *Jeffreycia*. We also thank Ingrid Pol-yin Lin of the Department of Botany for the scan of the isolectotype of *Vernonia brachycalyx* O. Hoffm. designated here as the neotype of *Vernonia abbotiana* O. Hoffm. Thanks also to the editor, Alexander Sennikov, for many careful observations and corrections.

## Supplementary Material

XML Treatment for Hoffmannanthus

XML Treatment for Hoffmannanthus abbotianus

XML Treatment for Jeffreycia

XML Treatment for Jeffreycia amaniensis

XML Treatment for Jeffreycia hildebrandtii

XML Treatment for Jeffreycia usambarensis

XML Treatment for Jeffreycia zanzibarensis

XML Treatment for Jeffreycia zeylanica
